# Using Behaviour Diagnostics to Identify Enablers and Barriers to Optimise Nurse and Midwife Manager Leadership Time

**DOI:** 10.1155/jonm/6498541

**Published:** 2025-03-27

**Authors:** Julie Considine, Philippa Blencowe, Naida Lumsden, Jordana Schlieff, Judy Currey

**Affiliations:** ^1^School of Nursing and Midwifery and Centre for Quality and Patient Safety in the Institute for Health Transformation, Deakin University, Geelong, Victoria 3220, Australia; ^2^Eastern Health, Box Hill, Victoria 3128, Australia

## Abstract

**Background:** Effective nursing and midwifery leadership benefits patients, staff and organisations. In February 2024, all nurse/midwife managers (*N* = 89) across one organisation transitioned to five allocated leadership days per week. For many nurse/midwife managers, whose default was to assume the clinical shift leader role when the unit was busy or short staffed, optimising use of five allocated leadership days per week required significant behaviour change.

**Aim:** The aims of this study were to: (i) examine the enablers and barriers to nurse/midwife managers using allocated leadership time to fulfil their core responsibilities and (ii) develop a theory-informed implementation plan to optimise allocated leadership time use.

**Methods:** A survey of all nurse/midwife managers, underpinned by the Theoretical Domains Framework, enabled identification of enablers and barriers to using allocated leadership time. The Behaviour Change Wheel was used to map enablers and barriers, identify intervention functions and behaviour change techniques to form an implementation plan. The APEASE criteria (acceptability, practicability, effectiveness, affordability, side effects/safety and equity) were applied to ensure effective and feasible strategies were selected.

**Results:** The response rate was 62.5% (55/89). Reflective motivation was the dominant enabler (clear goals, intentions and optimism). The most common barriers were reflective motivation (feeling responsible if an adverse event impacted staff or patients; perceptions of lack of control); automatic motivation (feelings of guilt, anxiety and stress if using allocated leadership time when their area is short staffed) and social opportunity (social influences and balancing the expectations of others). A range of intervention functions were necessary to support identified enablers and address identified barriers to nurse/midwife managers optimising their use of allocated leadership time.

**Conclusions:** Behaviour change theory is useful for identifying real-world enablers and barriers of nurse/midwife managers' use of allocated leadership time and developing a theory-informed implementation plan to optimise use of their allocated leadership time.

## 1. Introduction

Globally, effective nursing and midwifery leadership has clear benefits for patients, staff and organisations across a diversity of settings [1–4]. The nurse/midwife manager role is one of the most complex and valuable roles in healthcare [5]. Nurse/midwife managers devise and execute organisational initiatives and imperatives in complex clinical environments [5], and interface with patients and families; staff across all disciplines (nursing, medical, allied health and support staff) and hospital management [5]. Nurse/midwife managers have a unique role managing both human and capital resources [5], and linking professional and organisational logistics [6]. A key part of their role is achieving of both organisational and nursing/midwifery professional aims, which may sometimes be opposing [6].

There are several nursing leadership styles documented in the literature. Transactional leadership, focused on task assignment and completion, and characterised by reward or punishment, is a managerial, authoritative leadership style [7]. The suitability of transactional leadership for healthcare has been called into question in recent years and criticised for being task oriented and dis-empowering [8]. More contemporary leadership styles, particularly in nursing, are focused in growth of team members and include transformational leadership characterised by the elements of idealised influence, inspiration and motivation, intellectual stimulation and individualised consideration [9]; authentic leadership comprising self-awareness, internalised moral perspective, balanced processing and relational transparency [10]; servant leadership that is grounded in supporting other's professional growth through teamwork and shared decision making [10] and resonant leadership where leaders use their emotional intelligence to foster growth and engagement in others [4]. Recently, rebel leadership has emerged as a topic of inquiry and refers to nurse leadership that challenges the status quo with the intent of optimising patient care and grounded in the notion that nurses are critical to the healthcare system and have a deep understanding of patient needs [11].

Nurse/midwife manager roles of recruitment, retention and success planning are critical to patient safety, effective nursing and midwifery teams and organisational function. Studies spanning from United States of America, Canada, Europe, South Africa and Asia consistently show that the factors associated with increased nurse manager job satisfaction and retention including decision-making power and autonomy [12, 13]; job control [12, 13]; patient safety culture [12]; social support from managers, direct reports and interprofessional colleagues [12, 14]; organisational support [14] and meaningful work [13] increase nurse manager job satisfaction and retention. Causes of poor nurse manager job satisfaction and increased turnover include: high levels of job stress [12], overwhelming workloads [12, 14], poor supervisor relationships [14] and insufficient training or preparation for the nurse manager role [14]. The drivers of job satisfaction or dissatisfaction specifically for midwifery managers are not clearly reported in the published literature to date. To improve satisfaction and retention of nurse managers, institutions must cultivate practice environments that promote healthy workloads, strong interorganisational relationships, professional growth and success of their nurse managers [12–14].

In Australia, like many other countries, nurse/midwife managers are the largest critical mass of clinical leaders within an organisation; and their roles span acute care, subacute care, community and residential aged care settings. They are responsible for safety critical leadership such as: setting and enforcing the standard of clinical practices within their department; maintaining and monitoring performance against national accreditation standards; preventing and managing clinical risks; administrative functions (such as audits, reports, budgets); financial and environmental stewardship; legislative compliance such as staffing, workplace safety (physical and physiological); timely and effective patient access, flow and discharge; fostering patient and family/carer engagement in care; and staff recruitment, retention and development [5, 12].

To fulfil their leadership responsibilities, nurse/midwife managers require allocated leadership time. At one organisation in Melbourne Australia, all nurse/midwife managers were employed full time; however, the amount of allocated leadership time was variable, depending on the operational hours, budget magnitude and full-time equivalent staffing of the areas under their leadership, and this was true for both nurse and midwife managers. When clinical areas were short staffed, nurse/midwife managers often assumed the role of shift clinical leader. Whilst clinical support, mentoring and consultation are a component of nurse/midwife managers' roles, spending the entire day in a shift clinical leader capacity has adverse consequences of either undertaking their core leadership duties after hours, or falling behind in safety critical leadership responsibilities. Not fulfilling their leadership responsibilities creates stress and distress for nurse/midwife managers, whose retention in health services is critical to safe operations, nursing/midwifery and multi-disciplinary team cohesion, and patient safety.

The specific reasons influencing nurse/midwife managers use (or non-use) of allocated leadership time was poorly understood. Thus, a comprehensive understanding of the influences on nursing/midwifery managers use (or non-use) of allocated leadership time was needed to inform the organisation's strategic approach to supporting nurse/midwife managers. In February 2024, all nurse/midwife managers (*N* = 89) across the organisation transitioned to a full-time leadership role; that is, five allocated leadership days per week in recognition of the critical role that nurse/midwife managers play in patient safety, and to ensure all nurse/midwife managers had equitable working conditions. The primary intent of this transition was to enable nurse/midwife managers to improve consumer/patient satisfaction and clinical care through increased focus on safety and quality imperatives; support organisational readiness for short-notice accreditation visits; enhance recruitment and retention of clinical nurses and midwives to establish full staffing' and optimise nurse and midwife, and nurse/midwife manager well-being, engagement and job satisfaction. For many nurse/midwife managers, whose default was to assume the clinical shift leader role when the unit was busy or short staffed, transitioning to five allocated leadership days per week required significant behaviour change.

### 1.1. Aim

The aims of this study were to: (i) examine the enablers and barriers to nurse/midwife managers using allocated leadership time to fulfil their core responsibilities and (ii) develop a theory-informed implementation plan to optimise use of their allocated leadership time.

## 2. Methods

### 2.1. Design

This exploratory descriptive study had three components. First, enablers and barriers to using allocated leadership time were identified using a survey of all nurse/midwife managers. Second, these enablers and barriers were mapped to the Behaviour Change Wheel [15, 16], and intervention functions and behaviour change techniques were identified. Finally, feedback was sought from nurse/midwife managers regarding the recommended intervention functions and behaviour change techniques using the APEASE criteria (acceptability, practicability, effectiveness, affordability, side effects/safety and equity) [15]. The APEASE criteria were applied so that the implementation plan contained strategies most likely to be effective and feasible in this organisational context. This study was approved by the Human Research and Ethics Committee at Eastern Health (LR23-065-103462).

### 2.2. The Behaviour Change Wheel

**Figure 1:**
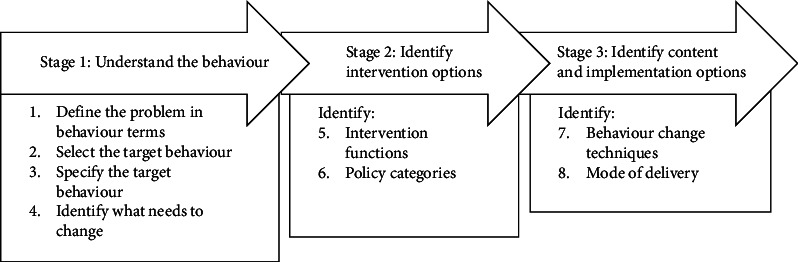
Behaviour change intervention design process.

The Behaviour Change Wheel is a synthesis of 19 frameworks of behaviour change and is underpinned by a model of behaviour called COM-B which stands for ‘capability', opportunity', ‘motivation' and behaviour [15, 16]. *Capability* refers to physical capability (dexterity, strength, sight) and psychological capability (knowledge, understanding); *Opportunity* refers to physical opportunity (environment, resources, time, triggers) and social opportunity (interpersonal influences, social norms, cultural norms) and *Motivation* consists of reflective motivation (conscious: want or need behaviour, care about consequences, aligned with goals & values, planning, beliefs about success) and automatic motivation (unconscious processes: habits, good or bad feelings, rewards or punishments) [15, 16]. Seven of the eight steps of behaviour change intervention design process from the Behaviour Change Wheel were used to guide this study (Figure 1) [15].

The problem (Step 1) was variability in nurse/midwife managers use of allocated leadership time. The target behaviour (Steps 2 and 3) is for nurse/midwife managers to optimise use of their allocated leadership time, including not assuming the role of shift leader or clinical nurse when there are staff shortages. The Theoretical Domains Framework (TDF) [17, 18] was used to identify what needs to change (Step 4). The TDF [17] is a synthesis of behavioural change theories that applies the science of intervention implementation in health care [17] to identify enablers and barriers to implementing change, and comprises 14 domains: (1) knowledge, (2) skills, (3) social/professional role and identity, (4) beliefs about capabilities, (5) optimism, (6) beliefs about consequences, (7) reinforcement, (8) intentions, (9) goals, (10) memory, attention and decision processes, (11) environment context and resources, (12) social influences, (13) emotion and (14) behavioural regulation.

The Behaviour Change Wheel was used to select interventions functions, which are broad categories of things that can change behaviour by changing the capability, opportunity and/or motivation to engage in the behaviour [15]. There are nine intervention functions (Education, Persuasion, Incentivisation, Coercion, Training, Enablement, Modelling, Environmental Restructuring and Restrictions) that are linked to the Behaviour Change Technique Taxonomy v1 [19], a theory-based taxonomy of 93 replicable behaviour change techniques [19]. Detailed descriptions of the Behaviour Change Wheel intervention functions and their relationship with the COM-B model are presented in Supporting Table 1 (Matrix of COM-B Components versus Intervention Functions). There are seven policy categories (Environmental/Social planning, Communication/Marketing, Legislation, Service provision, Regulation, Fiscal measures and Guidelines) that support the delivery of intervention functions [15]. Identification of policy categories was not undertaken for two reasons. First, the study setting was already governed by national and local policies that cover the Behaviour Change Wheel policy categories. Second, it was imperative that the intervention was suitable for implementation within the organisation's governance and policy framework. The Behaviour Change Techniques Taxonomy (v1) [19] was used to link the interventions functions identified in Step 5 to Behaviour Change Techniques (Step 7). Behaviour Change Techniques are considered the ‘active components' when designing an intervention aimed at behaviour change [15]. Finally, Step 8 was achieved using cognate knowledge of the research team and feedback from nurse/midwife managers.

### 2.3. Setting

The study setting was Eastern Health, Victoria, Australia. Eastern Health is one of Melbourne's largest metropolitan public health services with six hospitals offering bed-based services (four acute care, two subacute care, one planned surgical hospitals), and a range of day-based, community and ambulatory services. Maternity services are offered at two acute care sites, specialist clinics at four sites and multiple sites within the community. Eastern Health delivers over 1.4 million episodes of patient care annually [20]. Nurse and midwifery staffing in Victoria is governed by the *Safe Patient Care (Nurse and Midwife to Patient Ratios) Act* [21] that contains legislated nurse/midwife-to-patient ratios for different levels of hospital, and for different clinical areas; and is largely focused on bed-based (ward) services. At the time of undertaking this study, Eastern Health nursing and midwifery workforce data, like many other Victorian health services, showed workforce shortages were resulting in daily breaches of the Safe Patient Care Act ratios [22], meaning that nurses and midwives are caring for more patients than is legislated.

### 2.4. Participants

All nurse/midwife managers at Eastern Health's acute care, subacute care and community services (*N* = 89) were invited to complete an anonymous online survey about the enablers and barriers to using allocated leadership time in their clinical area. Managers from mental health and residential aged care were excluded as these areas function under different industrial and professional frameworks, which means their allocated leadership time is governed differently to nurse managers in the rest of the organisation. Consent was implied by completion and submission of the survey. At the time of the study, the number of days per week allocated as leadership time varied between nurse/midwife managers as follows: five days 42%, four days 35%, three days 13%, two days 4.5%, one day 2% and unknown 3.5%.

### 2.5. Data Collection

A survey based on the TDF domains [17] was developed by the research team, informed by the evidence to date regarding nurse and midwifery manager leadership, and observations of nurse and midwifery leadership behaviours at Eastern Health. Survey development using the TDF domains has been used in previous studies [23, 24]. The survey commenced with a section on participant characteristics (area of practice and years of experience in nursing or midwifery, nursing or midwifery management and nursing or midwifery management in current area). Thirty-four items were developed reflecting the TDF domains. Skills (related to physical capability) was the only domain not included as it was deemed that by virtue of appointment to the nurse or midwife manager role, they would have the requisite physical skills to perform their roles.

Validity is the extent to which an instrument measures what is it intended to measure [25]. To establish content and construct validity, the survey was constructed by an expert panel consisting of five organisational nursing and midwifery leaders, and three PhD-prepared nursing academics with expertise in clinical leadership, and behavioural and implementation science (including the senior author of previous published studies [23, 24] and Eastern Health's Professor of Nursing). The survey was then reviewed by members of the Nursing and Midwifery Professional Council (*n* = 16) comprising Directors of Nursing and Midwifery, and Directors of Learning & Teaching (*n* = 3). Face validity was established by piloting the survey with the aforementioned expert panel and three senior nurses from Eastern Health (one nursing research fellow and two nurse educators) who were ineligible to participate in the study.

The survey was hosted on Eastern Health's Research Electronic Data Capture (REDCap) [26] and distributed by the Professor of Nursing (who had no line management responsibilities for nurse or midwife managers) via email. Survey data were collected between 27 November and 8 December 2023. Quantitative results were mapped against the Behaviour Change Wheel [15, 16] domains of capability, opportunity and motivation to inform the most appropriate behaviour change interventions for successful implementation [16].

### 2.6. Data Analysis

Quantitative survey data were analysed using SPSS Version 29.0. Descriptive statistics were used to summarise the study data. The mean and standard deviation or the median and interquartile range were calculated for continuous data, and frequencies and percentages were calculated for categorical data. Survey items were considered enablers if they were positively worded with greater or equal to 70% agreement with a statement [24]. Items were considered barriers if they were positively worded with less than 70% agreement with a statement [24]. Negatively worded questions were reverse scored. Fisher's exact test was used to examine the relationship between enablers and barriers, and participant characteristics (years of experience classified as < 25^th^ percentile, 25–75^th^ percentile or > 75^th^ percentile; and organisational directorate).

## 3. Results

A total of 55 nurse/midwife managers (50 nurse and 5 midwifery managers) responded to the survey yielding a response rate of 62.5% (55/89). The small number of midwifery managers reflects Eastern Health's service delivery. Given the limited number of midwifery managers, and that all included managers function under the same professional and industrial frameworks, aside from management experience characteristics, the results will be presented as one group (nurse/midwife managers). The median years of professional and management experience is shown in Table 1. Participants were managers in the following organisational directorates: surgery including intensive care services (29.1%, *n* = 16), aged medicine (20.0%, *n* = 11), women's and children's (14.5%, *n* = 8), speciality medicine (12.7%, *n* = 7), acute medicine including emergency services (10.9%, *n* = 6) and other (12.7%, *n* = 7). Other included ambulatory care, medicine imaging, and specialist clinics.

### 3.1. Enablers and Barriers to Nurse/Midwife Managers Optimising Use of Leadership Time

There were 17 enablers and 19 barriers to optimising nurse/midwife managers' use of allocated leadership time with two survey items classified as both an enabler and barrier because the distributions were spread across positive and negative responses. The frequency of survey items coded as enablers and barriers are presented by the TDF domains and COM-B model in Table 2. Detailed coding is presented in Supporting Table 2 (Survey responses mapped to TDF and enabler or barrier classifications).

The strongest enablers were mostly related to reflective motivation (*n* = 12) but also spanned automatic motivation (*n* = 2), psychological capability (*n* = 2) and physical opportunity (*n* = 1) (Table 2). The TDF [17] domains only associated with enablers were: (i) knowledge, (ii) intentions, (iii) goals, (iv) optimism and (v) reinforcement: none of these domains were a barrier. The survey items that were the strongest enablers were beliefs that using leadership time will benefit: the organisation (100% agree with positive statement); patients (98.2% agree with positive statement) and staff (96.4% agree with positive statement); confidence in their role (98.2% agree with positive statement) and belief that they play a key role in patient safety (98.1% agree with positive statement) (Table 2).

The barriers were more diverse and spanned social opportunity (*n* = 5), reflective motivation (*n* = 5), automatic motivation (*n* = 4), physical opportunity (*n* = 3) and psychological capability (*n* = 2) (Table 2). The TDF [17] domains only associated with barriers were: (i) memory, attention and decision, (ii) behaviour regulation, (iii) social influences and (iv) emotions. The survey items that were the strongest barriers were the norm that when an area is short staffed, nurse or midwife managers take over running the shift (88.9% agree with negative statement); feelings of guilt if using leadership time when their area is short staffed (85.4% agree with negative statement) and running the shift is an automatic response during staff shortages (80.0% agree with negative statement) (Table 2).

The TDF [17] domains associated with both enablers and barriers were (i) social/professional role and identity, (ii) beliefs about consequences, (iii) beliefs about capabilities and (iv) environmental context and resources. Two survey items were coded as both enablers and barriers as they spanned both positive and negative responses: these were belief that it is the nurse/midwife manager's role to take over running the shift; and physical layout of the area making it difficult to use leadership time (Table 2).

Participants' years of nursing or midwifery management experience (categorised by < 25^th^ percentile, 25–75^th^ percentile and > 75^th^ percentile) had no effect on enablers or barriers to optimising nurse/midwife managers' use of allocated leadership time (Supporting Table 3: Survey responses coded as enablers or barrier per years of nursing or midwifery management experience). When participant's directorate was examined, there were only two items that had statistically significant differences (Supporting Table 4: Survey responses coded as enablers or barrier per participant's directorate). First, nurse and midwife managers in medical and surgical directorates were more likely to respond that a good understanding of the Safe Patient Care Act was an enabler than those in women's and children's or other directorates (48.8% vs. 31.7% vs. 9.8% vs. 9.8%, *p* = 0.006). Second, the believe that it is the nurse/midwife manager's role to take over running the shift when an area is short staffed was more likely to be an enabler (that is, participants disagreed) to optimising nurse/midwife managers' use of allocated leadership time for nurse and midwife managers in the medical and surgical directorates than those in women's and children's or other directorates (47.7% vs. 40% vs. 13.3% vs. 0.0%, *p* = 0.006) and less likely to be a barrier for nurse and midwife managers in women's and children's and surgical directorates than other or medical directorates (13.6% vs. 18.2% vs. 31.8% vs. 36.4%, *p* = 0.006).

### 3.2. Intervention Functions and Behaviour Change Techniques

The enablers and barriers were mapped to intervention functions according to their COM-B component (Table 3). All nine intervention functions were required to support identified enablers and address identified barriers to nurse/midwife managers optimising their use of leadership time.

Behaviour Change Techniques were mapped against the intervention functions, and enablers and barriers framed in the COM-B model [15, 16] using the Behaviour Change Techniques Taxonomy (v1) [19]. There were 32 Behaviour Change Techniques [15, 19] identified as being the most likely to be effective in addressing the enablers and barriers to nurse/midwife managers optimising use of leadership time: detailed mapping is presented in Supporting Table 3 (Enablers and barriers to optimising use of nurse and midwife manager leadership time and associated intervention functions and behaviour change techniques). The Behaviour Change Techniques [15, 19] were related to goals and planning; feedback and monitoring; social supports, rewards and incentives; restructuring social and physical environments; identity and self-belief; understanding consequences; comparison of behaviour and having information from credible source(s).

**Figure 2:**
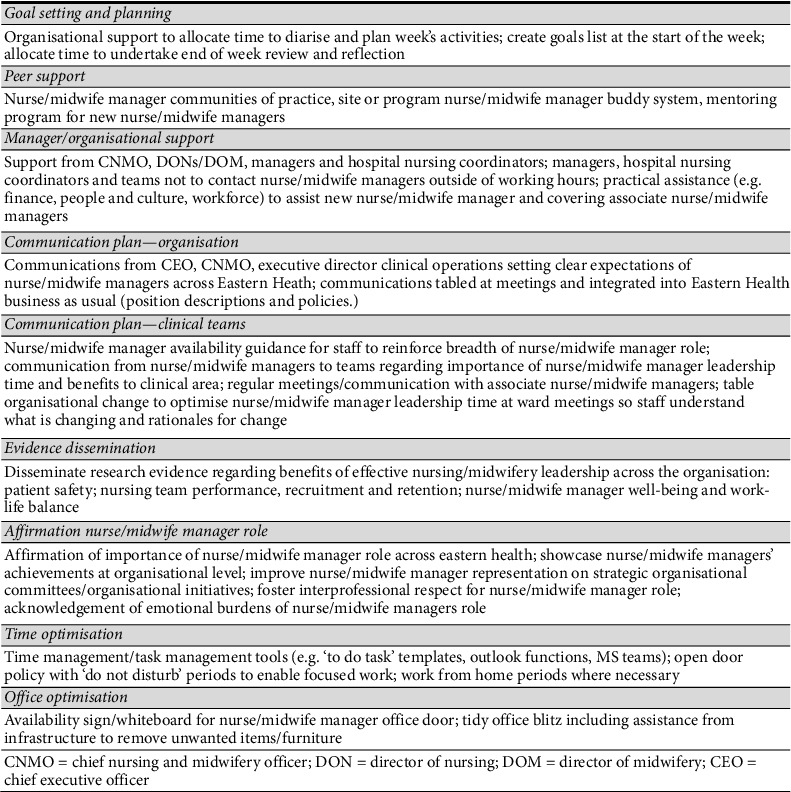
Implementation plan to optimise use of nurse/midwife manager leadership time.

**Figure 3:**
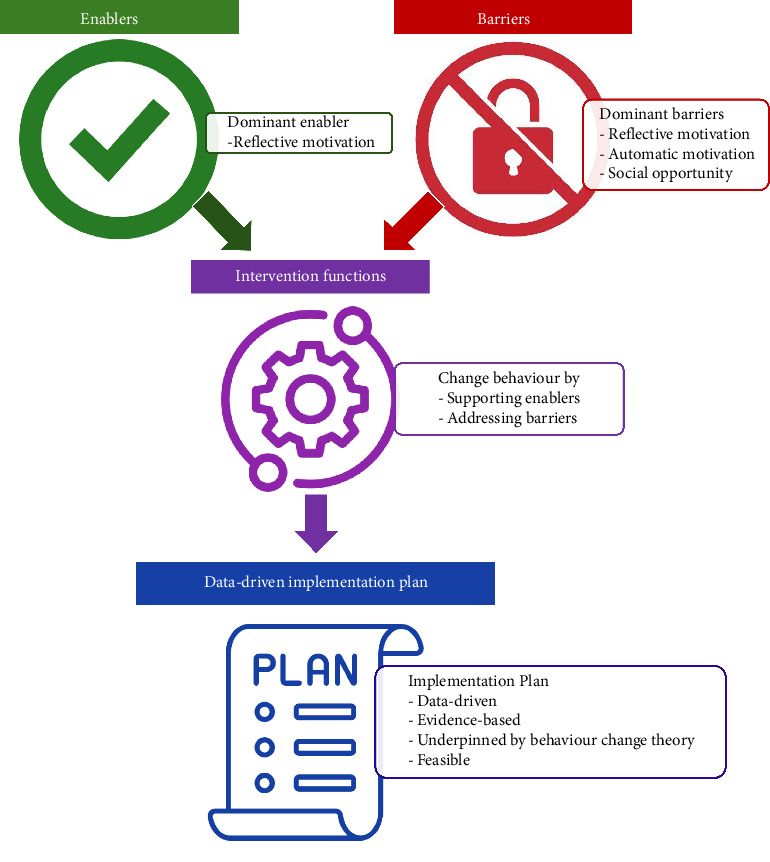
Summary of the study process and major findings.

In the final stage, the most appropriate mode(s) of delivery of each Behaviour Change Technique were determined by the research team and subject to the APEASE criteria in consultation with nurse/midwife managers to form an evidence-informed implementation plan (Figure 2). A summary of the study process and major findings is presented in Figure 3.

## 4. Discussion

This study had four major findings related to nurse/midwife managers optimising their use of leadership time. First, reflective motivation was the dominant enabler, and this was unaffected by years of management experience or directorate of employment. In behavioural terms, motivation encompasses internalised cognitive and emotional processes that influence behaviour [15, 27]. Reflective motivation comprises complex and conscious cognitive processes such as beliefs, goals and values; self-identity and beliefs about ability; wants or needs; care about consequences; making plans; and reflecting on and evaluating past events [15, 27]. The survey results highlighted that nurse/midwife managers had clear goals, intentions and optimism, and had optimistic beliefs about consequences of optimising leadership time for themselves, their teams and patients. Effective leadership, and particularly transformational leadership, enhances quality and safety of patient care [2, 28], patient-centred communication [3] and evidence-informed practice [3]; along with staff motivation [3], well-being [3] and job satisfaction [3, 4, 29, 30]. Study participants also trusted their teams of associate nurse/midwife managers (who are effectively second-in-charge) to manage shifts when their area was short staffed. Succession planning is vital to maintaining a critical mass of nurse/midwife managers, safeguarding operational continuity, ensuring team cohesiveness and function at the unit level, and containing costs related to nurse/midwife manager turnover [31, 32]. Providing associate nurse/midwife managers supported opportunity to manage shifts with high workloads or unplanned staff shortages is essential to their professional growth and progression to a nurse/midwife manager role.

Second, reflective and automatic motivation and social opportunity were the most common barriers and this was unaffected by years of management experience or directorate of employment. Whilst reflective motivation was a dominant enabler, feelings of responsibility in the case of an adverse event impacting staff or patients and perceptions of lack of control were areas of reflective motivation that were barriers to nurse/midwife managers remaining in their allocated leadership day roles. Automatic motivation involves unconscious processes such as habits, impulses, desires and inhibitions; good or bad emotional responses and feelings about rewards or punishments [15, 27]. The major barriers related to automatic motivation were related to emotion, specifically feelings of guilt, anxiety and stress if they were using allocated leadership time when their area is short staffed. The emotional burden of the nurse/midwife manager role is poorly understood. Studies of nurse managers' insights regarding their role highlighted issues related to the ‘emotional toll' and feelings of guilt when drawing boundaries with safety, such as not taking calls on days off [5], and guilt as a response to moral distress when efforts to advocate for their unit were ineffective [33]. The other dominant barrier was social influences and balancing the expectations of others: clinical teams, hospital nurse coordinators and the organisation. Participants from qualitative studies described the nurse manager role as ‘…the toughest position on the planet' with no one between the nurse manager and their staff, patients, families and hospital administration [5], and feeling ‘…responsible for everything… 24/7' as the only link between their clinical area and hospital administration [33].

Nurse/midwife manager relationship with their clinical team is important, but there needs to be mutual understanding of each other's role demands. Some studies highlight a disconnect between nurses' perceptions of the nurse manager role and the actual nurse manager role [34, 35], and many nurses do not connect nurse/midwife manager's daily tasks, and their on-shift experience [34]. Expectations that nurse managers maintain the same level of clinical competence as direct care nurses/midwives, and be available to provide direct care when the unit is busy, highlights a lack of understanding of the nurse/midwife manager's role [34]. Further expectations regarding nurse/midwife managers' presence and visibility may be unrealistic given their other role demands [34]. Although many nurse/midwife managers do not provide direct patient care, having meaningful work focused on positive patient outcomes, empowering staff and creating a positive work environment is important to nurse/midwife managers and increases their own retention [13]. Similarly, a positive relationship with their manager or supervisor such as manager support or transformational supervisor leadership behaviours increases nurse/midwife manager work satisfaction, decreases intention to leave and increases retention [13]. Unclear authority from supervisors and unclear organisational directives are associated with increased nurse/midwife manager turnover [13].

Third, a range of intervention functions were necessary to support identified enablers and address identified barriers to nurse/midwife managers optimising their use of allocated leadership time, irrespective of years of management experience or directorate of employment. The lack of significant difference in survey responses between experienced versus less experienced managers and managers working in different operational directorates suggests that enablers and barriers to optimising their use of allocated leadership time transcend context and personal attributes. A 2024 scoping review of 18 studies of acute health care leaders (11 of which were situated in nursing), showed that the personal and organisational attributes supporting transformational leadership in health care are not well understood and warrant further evaluation [36].

Using the COM-B model and TDF to develop implementation strategies can achieve successful and sustained behaviour change in complex clinical environments [37, 38], and the TDF is also helpful when designing behaviour substitution interventions aimed at de-implementation of current behaviours [39]. Results of this study highlight the complexity of barriers to nurse/midwife managers optimising use of allocated leadership time. The usual defaults of education, training and directives to nurse/midwife managers, when mapped to real-life barriers, have no or limited effect. For example, barriers related to physical and social opportunity require solutions such as environmental restructuring and managing people other than the nurse/midwife managers such as clinical teams and supervisors [15, 16]. Barriers related to reflective and automatic motivation require interventions that resolve internalised cognitive and emotional processes drivers of behaviour such as persuasion, incentives, modelling and enablement [15, 16, 27]. Future leadership development initiatives should include participant-specific barriers and enablers assessments so that the approach is based on real-world data and strategies can be tailored to cohort-specific needs. Using agenda-led outcome-based analysis (ALOBA) in small group leadership training programs is recommended to address individual learning needs and provide tailored feedback that is practical and impactful [40]. For clinicians with a growth mindset, and in an established psychologically safe space, ALOBA has been used successfully to address verbal and non-verbal behaviours in conversations successfully for decades [40].

Finally, development of a data-driven, evidence-based implementation plan to optimise use of nurse and midwife manager leadership time was possible through use of behaviour change theory. Achieving sustained behaviour change in healthcare is complex [41] and it is well documented that behaviour change interventions are more effective when underpinned by psychological or behaviour change theory [42]. The BCW [15, 16], including the TDF and Behaviour Change Technique Taxonomy v1 [19], provides a framework to assess enablers and barriers of behaviour change, and make data-driven decisions about what needs to happen for effective behaviour change. The APEASE criteria [15] enable systematic decision making and prioritisation of behaviour change techniques and intervention functions, and can be used to methodologically assess intervention acceptability and feasibility. Implementation outcomes and sustainability are important considerations, although beyond the scope of the work presented in this paper. Again, a theoretical approach such as the RE-AIM framework (Reach, Effectiveness, Adoption, Implementation and Maintenance/Sustainability) provides rigorous and robust methods for evaluating implementation outcomes and sustainability [43, 44].

### 4.1. Limitations

There are a number of limitations that should be considered when interpreting the study findings. First this study occurred at one healthcare organisation that may have differing characteristics to other organisations: the implementation plan may not be generalisable to other organisations, however, the process of using a theory-driven approach could be applied to other organisations and contexts of care. The voluntary nature of survey completion may be a source of sampling bias but the response rate was 62.5% so more nurse/midwife managers responded than not. The modest sample size may have made subanalyses related to organisational directorate and manager experience prone to type-II error. Finally, there is a risk of social-desirability bias, whereby participants answered questions in a favourable manner. Given the spread of positive and negative responses, the authors do not think this influenced the study findings.

## 5. Conclusions

Behaviour change theory is useful for identifying real-world enablers and barriers of nurse/midwife managers' use of allocated leadership time and developing a theory-informed implementation plan to optimise use of their allocated leadership time. Future work will focus on determining the effectiveness of implementation of allocated nurse/midwife manager leadership time, and monitoring and addressing emerging barriers to leadership time use.

## Figures and Tables

**Table 1:** Years of experience and management experience.

Years of experience	*n* ^#^	Median	Interquartile range
Professional experience			
• Nursing	54	20.5	15.0–31.7
• Midwifery	7	12.0	8.0–16.0
Management experience			
• Nursing	52	7.0	3.6–12.7
• Midwifery	6	3.0	2.0–5.5
Management experience in the current clinical area			
• Nursing	50	5.5	3.0–9.0
• Midwifery	6	2.0	1.75–3.5

^#^All registered midwife participants were also registered nurses.

**Table 2:** Frequency of survey items coded as barriers and enablers by theoretical domains, framework domains and COM-B model (capability, opportunity and motivation = behaviour).

COM-B classification and TDF domains	Number of survey items coded as enablers	Number of survey items coded as barriers
Capability: physical		
• Skills	0	0
Capability: psychological	2	2
• Knowledge	2	0
• Memory, attention and decision processes	0	1
• Behavioural regulation	0	1
Opportunity: physical	1	2
• Environmental context and resources	1	3
Opportunity: social	0	5
• Social influences	0	5
Motivation: reflective	12	5
• Beliefs about consequences	3	2
• Optimism	3	0
• Social/professional role and identity	2	1
• Beliefs about capabilities	2	2
• Intentions	1	0
• Goals	1	0
Motivation: automatic	2	4
• Reinforcement	2	0
• Emotion	0	4
Total	17	19

Abbreviation: TDF = theoretical domains framework.

**Table 3:** Matrix of COM-B components by enablers and barriers versus intervention functions.

COM-B components		Intervention functions								
		Education	Persuasion	Incentives	Coercion	Training	Restriction	Environmental restructuring	Modelling	Enablement
*Enablers*	*n* (survey items)									
Capability: psychological	2	✓				✓				✓
Opportunity: physical	1					✓	✓	✓		✓
Motivation: reflective	12	✓	✓	✓	✓					
Motivation: automatic	2		✓	✓	✓	✓		✓	✓	✓

*Barriers*	*n* (survey items)									
Capability: psychological	2	✓				✓				✓
Opportunity: physical	3					✓	✓	✓		✓
Opportunity: social	5						✓	✓	✓	✓
Motivation: reflective	5	✓	✓	✓	✓					
Motivation: automatic	4		✓	✓	✓	✓		✓	✓	✓

✓ = likely to be effective.

## Data Availability

The data that support the findings of this study are available upon request from the corresponding author. The data are not publicly available due to privacy or ethical restrictions.

## Supporting Information

In Supporting Table 1, detailed descriptions of the Behaviour Change Wheel intervention functions and their relationship with the COM-B model are provided.

In Supporting Table 2, detailed mapping of the frequency of survey items coded as enablers and barriers by the TDF domains and COM-B is presented.

In Supporting Table 3, the frequency of survey items coded as enablers and barriers by participant's years of nursing or midwifery management experience is presented.

In Supporting Table 4, the frequency of survey items coded as enablers and barriers by participant's organisational directorate is presented.
