# Supplementary material for: Using Behaviour Diagnostics to Identify Enablers and Barriers to Optimise Nurse and Midwife Manager Leadership Time
Source: J Nurs Manag. 2025 Mar 27;2025:6498541. doi: 10.1155/jonm/6498541 (PMC11968169; doi:10.1155/jonm/6498541)
Supplement: Supporting Information — Additional supporting information can be found online in the Supporting Information section. [file 6498541.f1.pdf]

Supplementary Table 1: Matrix of COM-B Components versus Intervention Functions

COM-B Components	Intervention functions*								
	Education	Persuasion	Incentives	Coercion	Training	Restriction	Environmental restructuring	Modelling	Enablement
Capability: Physical					■				■
Capability: Psychological	■				■				■
Opportunity: Physical					■	■	■		■
Opportunity: Social						■	■	■	■
Motivation: Automatic		■	■	■	■		■	■	■
Motivation: Reflective	■	■	■	■					

*'Intervention functions' are broad categories of things that can change behaviour and are designed to capability, opportunity and/or motivation resulting in behaviour change.

- Education: Increasing knowledge and understanding
- Persuasion: Using communication to create positive or negative feelings to stimulate action.
- Incentivisation: Creating an expectation of a reward
- Coercion: Creating an expectation of punishment
- Training: Increasing psychological or physical skills
- Restriction: Using rules to constraining behaviour
- Environmental restructuring: Constraining or promoting behaviour by changing the physical or social environment
- Modelling: Showing examples of the behaviour for people to aspire to
- Enablement: Providing support, increasing means and reducing barriers to increase capability (beyond education and training) or opportunity (beyond environmental restructuring)

Supplementary Table 2: Survey responses mapped to Theoretical Domains Framework and enabler or barrier classifications

**Survey items**<br>** ** negatively worded question = reverse scored	**Negative (%)**	**Neutral (%)**	**Positive (%)**	**TDF Domain**	**COM-B**	**Enabler or barrier**
**Nurse / Midwife Manager role**						
I feel confident as a Nurse/Midwife Manager	1.8	0	98.2	Beliefs: capabilities	M:R	Enabler
I believe that Nurse/Midwife Managers play a key role in patient safety	0	1.8	98.1	Beliefs: capabilities	M:R	Enabler
I have control over my role as a Nurse/Midwife Manager	32.7	3.6	63.6	Beliefs: capabilities	M:R	Barrier
When I come onto a shift, I always have a clear idea of what I need to achieve for the day	23.6	1.8	74.6	Goals	M:R	Enabler
I have a good understanding of the Safe Patient Care Act[#]	10.9	5.5	83.6	Knowledge	C:Ps	Enabler
I am able to recognise when there is a breach of the Safe Patient Care Act[#]	5.5	5.5	74.5	Knowledge	C:Ps	Enabler
** If my area has Safe Patient Care Act breaches, I will be punished[#]	56.4	7.3	12.7	Reinforcement	M:A	Enabler
** If my area has Safe Patient Care Act breaches, my team will be punished[#]	54.6	7.3	14.6	Reinforcement	M:A	Enabler
**Managing staffing deficits**						
** When an area is short staffed, I believe it is the Nurse/Midwife Manager's role to take over running the shift	54.6	5.5	40.0	Social/Professional Role & Identity	M:R	Enabler / Barrier
** When an area is short staffed, at Eastern Health, it is normal for Nurse Managers / Midwife Managers to take over running the shift	7.3	1.8	88.9	Social Influences	O:S	Barrier
** When my area is short staffed, taking over running the shift is an automatic response	20.0	0	80.0	Behaviour regulation	C:Ps	Barrier
When my area is short staffed, I trust the Associate Nurse Manager/Associate Midwife Manager or shift leader to run the shift	1.8	1.8	96.3	Social / Professional Role & Identity	M:R	Enabler
** When my area is short staffed, the nursing coordinators expect me to take over running the shift	10.9	10.9	78.2	Social Influences	O:S	Barrier
** When my area is short staffed, my team expect me to take over running the shift	18.2	3.6	78.1	Social Influences	O:S	Barrier
**Leadership time / management days**						
When I am on leadership time, I always intend to use that time for Nurse/Midwife Manager tasks	5.4	3.6	90.9	Intentions	M:R	Enabler
If I use my leadership time, I can make things better for patients	0	1.8	98.2	Optimism	M:R	Enabler
If I use my leadership time, I can make things better for staff	1.8	0	96.4	Optimism	M:R	Enabler
If I use my leadership time, I can make things better for Eastern Health	0	0	100.0	Optimism	M:R	Enabler
I believe that me using my leadership time increases patient safety	1.8	3.6	94.5	Beliefs: consequences	M:R	Enabler
I believe that me using my leadership time increases staff well-being	3.6	5.5	90.9	Beliefs: consequences	M:R	Enabler
I believe that me using my leadership time increases my well-being	3.6	3.6	92.8	Beliefs: consequences	M:R	Enabler
When my area is short staffed, I am able to make decisions about team nursing and patient allocations that enable me to use my leadership time	38.2	5.5	56.3	Memory, Attention & Decisions	C:Ps	Barrier
When my area is short staffed, Eastern Health supports me to use my leadership time	49.1	5.5	45.4	Social Influences	O:S	Barrier
When my area is short staffed, I am able to reconfigure nurse / midwife staffing to enable me to use my leadership time	52.7	1.8	45.4	Beliefs: capabilities	M:R	Barrier
When my area is short staffed, I would be more likely to use my leadership time if I knew other Nurse Managers/Midwife Managers did the same	27.3	38.2	32.7	Social Influences	O:S	Barrier
** When my area is short staffed, if I am in the office using my leadership time, I feel guilty	9.1	5.5	85.4	Emotion	M:A	Barrier

**Survey items**<br>** ** negatively worded question = reverse scored	**Negative (%)**	**Neutral (%)**	**Positive (%)**	**TDF Domain**	**COM-B**	**Enabler or barrier**
**Leadership time / management days (continued)**						
** When my area is short staffed, if I am in the office using my leadership time, I feel anxious	21.8	10.9	67.3	Emotion	M:A	Barrier
** When my area is short staffed, if I am in the office using my leadership time, I feel stressed	14.5	7.3	78.2	Emotion	M:A	Barrier
When my area is short staffed, if I am in the office using my leadership time, I feel calm	74.6	9.1	16.4	Emotion	M:A	Barrier
** When my area is short staffed, if I am in the office using my leadership time, I would feel responsible if something bad happens to a patient	18.2	3.6	78.2	Beliefs: consequences	M:R	Barrier
** When my area is short staffed, if I am in the office using my leadership time, I would feel responsible if something bad happens to a staff member	14.5	3.6	81.8	Beliefs: consequences	M:R	Barrier
**Work environment**						
** The physical layout of my area makes using my leadership time difficult	43.7	14.5	41.8	Environment	O:P	Enabler / Barrier
My office is a pleasant working environment	23.6	16.4	60.0	Environment	O:P	Barrier
I have everything I need in my office	25.4	10.9	63.6	Environment	O:P	Barrier

Legend: TDF = Theoretical Domains Framework; C = capability, C: Ps = physiological capability; C:Ph = physical capability; O = opportunity, O:P = physical opportunity; O:S = social opportunity, M = motivation, M:A = automatic motivation; M:R = reflective motivation; B = behaviour
[#] Safe Patient Care Act did not apply to 8 nurse / midwife managers

Supplementary Table 3: Survey responses coded as enablers or barrier per participant's years of nursing or midwifery management experience

Survey items<br>** ** negatively worded question = reverse scored	Enablers				Barriers				p*
	Total (n)	Q1 (%)	Q2-3 (%)	Q4 (%)	Total (n)	Q1 (%)	Q2-3 (%)	Q4 (%)	
**Nurse / Midwife Manager role**									
I feel confident as a Nurse/Midwife Manager	54	27.8	48.1	24.1	1	100.0	0.0	0.0	0.527
I believe that Nurse/Midwife Managers play a key role in patient safety	0	N/A	N/A	N/A	54	29.6	46.3	24.1	N/A
I have control over my role as a Nurse/Midwife Manager	35	31.4	42.9	25.7	18	27.8	55.6	16.7	0.752
When I come onto a shift, I always have a clear idea of what I need to achieve for the day	41	24.4	48.8	26.8	13	38.5	46.2	15.4	0.552
I have a good understanding of the Safe Patient Care Act[#]	46	23.9	50.0	26.1	6	33.3	50.0	16.7	1.000
I am able to recognise when there is a breach of the Safe Patient Care Act[#]	41	22.0	53.7	24.4	3	33.3	66.7	0.0	1.000
** If my area has Safe Patient Care Act breaches, I will be punished[#]	31	29.0	38.7	32.3	7	14.3	85.7	0.0	0.106
** If my area has Safe Patient Care Act breaches, my team will be punished[#]	30	27.8	48.1	24.1	8	25.0	75.0	0.0	0.527
**Managing staffing deficits**									
** When an area is short staffed, I believe it is the Nurse/Midwife Manager's role to take over running the shift	30	20.0	56.7	23.3	22	40.9	36.4	22.7	0.131
** When an area is short staffed, at Eastern Health, it is normal for Nurse Managers / Midwife Managers to take over running the shift	4	50.0	50.0	0.0	50	28.0	46.0	26.0	0.219
** When my area is short staffed, taking over running the shift is an automatic response	11	18.2	45.5	36.4	44	31.8	47.7	20.5	0.563
When my area is short staffed, I trust the Associate Nurse Manager/Associate Midwife Manager or shift leader to run the shift	53	28.3	47.2	24.5	1	100.0	0.0	0.0	0.517
** When my area is short staffed, the nursing coordinators expect me to take over running the shift	6	33.3	33.3	33.3	43	32.6	46.5	20.9	0.537
** When my area is short staffed, my team expect me to take over running the shift	10	10.0	70.0	20.0	43	30.2	44.2	25.6	0.855
**Leadership time / management days**									
When I am on leadership time, I always intend to use that time for Nurse/Midwife Manager tasks	50	30.0	44.0	26.0	3	33.3	66.7	0.0	0.787
If I use my leadership time, I can make things better for patients	0	N/A	N/A	N/A	54	27.8	48.1	24.1	N/A
If I use my leadership time, I can make things better for staff	53	30.2	45.3	24.5	1	0.0	100.0	0.0	1.000
If I use my leadership time, I can make things better for Eastern Health	0	N/A	N/A	N/A	55	29.1	47.3	23.6	N/A
I believe that me using my leadership time increases patient safety	52	28.8	46.2	25.0	1	0.0	100.0	0.0	1.000
I believe that me using my leadership time increases staff well-being	50	26.0	48.0	26.0	2	50.0	50.0	0.0	1.000
I believe that me using my leadership time increases my well-being	51	25.5	49.0	25.5	2	100.0	0.0	0.0	0.133
When my area is short staffed, I am able to make decisions about team nursing and patient allocations that enable me to use my leadership time	31	35.5	41.9	22.6	21	14.3	57.1	28.6	0.271
When my area is short staffed, Eastern Health supports me to use my leadership time	25	28.0	48.0	24.0	27	29.6	44.4	25.9	1.000
When my area is short staffed, I am able to reconfigure nurse / midwife staffing to enable me to use my leadership time	24	29.2	50.0	20.8	29	24.1	48.3	27.6	0.682
When my area is short staffed, I would be more likely to use my leadership time if I knew other Nurse Managers/Midwife Managers did the same	18	27.8	50.0	22.2	15	20.0	66.7	13.3	0.705
** When my area is short staffed, if I am in the office using my leadership time, I feel guilty	5	20.0	80.0	0.0	47	31.9	40.4	27.7	0.356

**Leadership time / management days (continued)**									
** When my area is short staffed, if I am in the office using my leadership time, I feel anxious	12	33.3	41.7	25.0	37	27.0	51.4	21.6	0.832
** When my area is short staffed, if I am in the office using my leadership time, I feel stressed	8	25.0	62.5	12.5	43	27.9	44.2	27.9	0.709
When my area is short staffed, if I am in the office using my leadership time, I feel calm	9	22.2	66.7	11.1	41	29.3	43.9	26.8	0.573
** When my area is short staffed, if I am in the office using my leadership time, I would feel responsible if something bad happens to a patient	10	30.0	50.0	20.0	43	30.2	44.2	25.6	1.000
** When my area is short staffed, if I am in the office using my leadership time, I would feel responsible if something bad happens to a staff member	8	37.5	37.5	25.0	45	28.9	46.7	24.4	0.888
**Work environment**									
** The physical layout of my area makes using my leadership time difficult	24	20.8	54.2	25.0	23	43.5	39.1	17.4	0.246
My office is a pleasant working environment	33	33.3	45.5	21.2	13	15.4	61.5	23.1	0.475
I have everything I need in my office	35	31.4	42.9	25.7	14	28.6	64.3	7.1	0.348

Q1 = <25th percentile years of experience (n=16); Q2-3 = 25th – 75th percentile years of experience (n=26); Q4 = >75th percentile years of experience (n=13)
[#] Safe Patient Care Act did not apply to 8 nurse / midwife managers
B = barrier; E = enabler; * Fisher's exact test

Supplementary Table 4: Survey responses coded as enablers or barrier per participant's directorate

Survey items<br>** negatively worded question = reverse scored	Enablers					Barriers					p*
	Total (n)	Med (%)	Surg (%)	W&C (%)	Other (%)	Total (n)	Med (%)	Surg (%)	W&C (%)	Other (%)	
**Nurse / Midwife Manager role**											
I feel confident as a Nurse/Midwife Manager	54	44.4	27.8	14.8	13.0	1	0.0	100.0	0.0	0.0	0.564
I believe that Nurse/Midwife Managers play a key role in patient safety	54	44.4	27.8	14.8	13.0	0	N/A	N/A	N/A	N/A	N/A
I have control over my role as a Nurse/Midwife Manager	35	45.7	25.7	11.4	17.1	18	38.9	38.9	16.7	5.6	0.550
When I come onto a shift, I always have a clear idea of what I need to achieve for the day	41	36.6	36.6	12.2	14.6	13	61.5	7.7	23.1	7.7	0.119
I have a good understanding of the Safe Patient Care Act#	41	48.8	31.7	9.8	9.8	6	0.0	33.3	50.0	16.7	**0.006**
I am able to recognise when there is a breach of the Safe Patient Care Act#	41	48.8	31.7	9.8	9.8	3	0.0	33.3	33.3	33.3	0.087
** If my area has Safe Patient Care Act breaches, I will be punished#	31	45.2	29.0	16.1	9.7	7	71.4	28.6	0.0	0.0	0.789
** If my area has Safe Patient Care Act breaches, my team will be punished#	30	43.3	30.0	16.7	10.0	8	75.0	25.0	0.0	0.0	0.456
**Managing staffing deficits**											
** When an area is short staffed, I believe it is the Nurse/Midwife Manager's role to take over running the shift	30	46.7	40.0	13.3	0.0	22	36.4	18.2	13.6	31.8	**0.006**
** When an area is short staffed, at Eastern Health, it is normal for Nurse Managers / Midwife Managers to take over running the shift	4	25.0	50.0	25.0	0.0	50	46.0	26.0	14.0	14.0	0.616
** When my area is short staffed, taking over running the shift is an automatic response	11	45.5	36.4	18.2	0.0	44	43.2	27.3	13.6	15.9	0.633
When my area is short staffed, I trust the Associate Nurse Manager/Associate Midwife Manager or shift leader to run the shift	53	45.3	30.2	13.2	11.3	1	0.0	0.0	100.0	0.0	0.259
** When my area is short staffed, the nursing coordinators expect me to take over running the shift	6	33.3	33.3	33.3	0.0	43	46.5	30.2	14.0	9.3	0.571
** When my area is short staffed, my team expect me to take over running the shift	10	50.0	30.0	10.0	10.0	43	41.9	27.9	16.3	14.0	1.000
**Leadership time / management days**											
When I am on leadership time, I always intend to use that time for Nurse/Midwife Manager tasks	50	46.0	28.0	14.0	12.0	3	0.0	66.7	33.3	0.0	0.216
If I use my leadership time, I can make things better for patients	54	42.6	29.6	14.8	13.0	0	N/A	N/A	N/A	N/A	N/A
If I use my leadership time, I can make things better for staff	54	42.6	29.6	14.8	13.0	1	0.0	0.0	0.0	100.0	0.130
If I use my leadership time, I can make things better for Eastern Health	55	43.6	29.1	14.5	12.7	0	N/A	N/A	N/A	N/A	N/A
I believe that me using my leadership time increases patient safety	52	42.3	30.8	15.4	11.5	1	100.0	0.0	0.0	0.0	1.000
I believe that me using my leadership time increases staff well-being	50	42.0	32.0	16.0	10.0	2	50.0	0.0	0.0	50.0	0.428
I believe that me using my leadership time increases my well-being	51	45.1	31.4	13.7	9.8	2	0.0	0.0	50.0	50.0	0.066
When my area is short staffed, I am able to make decisions about team nursing and patient allocations that enable me to use my leadership time	31	45.2	29.0	16.1	9.7	21	47.6	28.6	9.5	14.3	0.926
When my area is short staffed, Eastern Health supports me to use my leadership time	25	48.0	24.0	20.0	8.0	27	37.0	37.0	11.1	14.8	0.559

Survey items<br>** negatively worded question = reverse scored	Enablers					Barriers					p*
	Total (n)	Med (%)	Surg (%)	W&C (%)	Other (%)	Total (n)	Med (%)	Surg (%)	W&C (%)	Other (%)	
**Leadership time / management days (continued)**											
When my area is short staffed, I am able to reconfigure nurse / midwife staffing to enable me to use my leadership time	25	48.0	20.0	16.0	16.0	29	41.4	34.5	13.8	10.3	0.716
When my area is short staffed, I would be more likely to use my leadership time if I knew other Nurse Managers/Midwife Managers did the same	18	50.0	22.2	11.1	16.7	15	40.0	33.3	26.7	0.0	0.314
** When my area is short staffed, if I am in the office using my leadership time, I feel guilty	5	20.0	60.0	20.0	0.0	47	44.7	27.7	12.8	14.9	0.433
** When my area is short staffed, if I am in the office using my leadership time, I feel anxious	12					37					1.000
** When my area is short staffed, if I am in the office using my leadership time, I feel stressed	8	41.7	25.0	16.7	16.7	43	37.8	32.4	16.2	13.5	0.196
When my area is short staffed, if I am in the office using my leadership time, I feel calm	9	62.5	12.5	0.0	25.0	41	34.9	34.9	18.6	11.6	0.132
** When my area is short staffed, if I am in the office using my leadership time, I would feel responsible if something bad happens to a patient	10	44.4	11.1	11.1	33.3	43	39.0	36.6	17.1	7.3	0.776
** When my area is short staffed, if I am in the office using my leadership time, I would feel responsible if something bad happens to a staff member	8	30.0	20.0	40.0	10.0	45	46.5	27.9	14.0	11.6	0.884
**Work environment**											
** The physical layout of my area makes using my leadership time difficult	24	37.5	25.0	25.0	12.5	23	44.4	31.1	13.3	11.1	0.636
My office is a pleasant working environment	33	33.3	33.3	16.7	16.7	13	52.2	26.1	13.0	8.7	0.056
I have everything I need in my office	35	51.5	24.2	18.2	6.1	14	23.1	38.5	7.7	30.8	0.782

Med (n=24) = general medicine, aged medicine, speciality medicine and emergency services; Surg (n=16) = general surgery, speciality surgery and intensive care services; W&C (n=8) = women's and children's; Other (n=7) = ambulatory, medical imaging, specialist clinics
[#] Safe Patient Care Act did not apply to 8 nurse / midwife managers
B = barrier; E = enabler; * Fisher's exact test
